# Time-Series Associations between Public Interest in COVID-19 Variants and National Vaccination Rate: A Google Trends Analysis

**DOI:** 10.3390/bs12070223

**Published:** 2022-07-09

**Authors:** Cecilia Cheng

**Affiliations:** Social and Health Psychology Laboratory, Department of Psychology, The University of Hong Kong, Pokfulam, Hong Kong, China; ceci-cheng@hku.hk

**Keywords:** information seeking, search query, infodemiology, infosurveillance, coping, pandemic anxiety

## Abstract

The emergence of a constantly mutating novel virus has led to considerable public anxiety amid the COVID-19 pandemic. Information seeking is a common strategy to cope with pandemic anxiety. Using Google Trends analysis, this study investigated public interest in COVID-19 variants and its temporal associations with the disease-prevention measure of vaccination during the initial COVID-19 vaccine rollout period (13 December 2020 to 25 September 2021). Public interest was operationalized as the relative search volume of online queries of variant-related terms in the countries first affected by the Alpha, Beta, and Delta variants: the UK, South Africa, and India, respectively. The results show that public interest in COVID-19 variants was greater during the Delta-variant-predominant period than before this period. The time-series cross-correlation analysis revealed positive temporal associations (i.e., greater such public interest was accompanied by an increase in national vaccination rate) tended to occur more frequently and at earlier time lags than the negative temporal associations. This study yielded new findings regarding the temporal changes in public interest in COVID-19 variants, and the between-country variations in these public interest changes can be explained by differences in the rate and pace of vaccination among the countries of interest.

## 1. Introduction

Since early 2020, the rapid evolution of coronavirus disease 2019 (COVID-19) has led to a global health crisis, transforming the lives of people worldwide into a “new normal” [1,2]. COVID-19 is caused by infection with severe acute respiratory syndrome coronavirus 2 (SARS-CoV-2), which is highly transmissible [3]. Since the onset of the COVID-19 pandemic, health anxiety has escalated throughout the world [4,5,6,7], and many people are concerned about themselves and their family members contracting COVID-19 [8,9,10].

As relatively little is known about COVID-19 etiology, treatment, and preventive measures, many people have coped with the crisis by seeking information on the Internet to make sense of the outbreak and to understand more about this novel virus [11,12,13]. Such surges in coping through information seeking among the general public can be explained by the uncertainty theory of anxiety, which postulates anxiety as an “epistemic” emotion that is related to the generation of knowledge and perception of knowledge quality [14]. According to this theory, individuals tend to feel anxious when in new or unfamiliar circumstances. Their epistemic needs become stronger, and they are motivated to predict future events in order to minimize or eliminate possible threats. Seeking out information is a major way to gratify heightened epistemic needs and mitigate anxious feelings, and in the current era, information seeking is widely conducted on the Internet [15].

### 1.1. Infodemiology in the Context of the COVID-19 Pandemic

Among social, communication, and health scientists, there is a growing research interest in infodemiology, which advocates the utilization of population-level Web-based data for explaining and predicting health behaviors and epidemics [16]. With the advancement of information technology (e.g., search engines, social networking sites), online search queries and social media posts constitute major information sources for infodemiological research [17]. Infodemiological data can provide valuable insights into population behavior and serve as predictors to identify and forecast trends and incidences of emerging diseases (e.g., infections, death tolls). Findings drawn from this innovative approach serve as catalysts for public policy formulation and reforms for enhancing societal wellness [16]. The importance of this novel approach has been recently recognized by the World Health Organization (WHO), with their first infodemiology conference held in 2020.

Previous studies have used infodemiological methods to reveal public interest in particular topics during the initial wave of the COVID-19 pandemic. For instance, there were sudden surges in Internet search volumes for query terms such as “*coronavirus symptom*” and “*hand sanitizer*” on the day following the announcement of the first COVID-19 confirmed case in various states in the US [18]. Population-level interest in understanding the etiology of the novel virus was reflected in similar surges in Internet search volumes for the query terms “*bat*” and “*pangolin*” [19], both of which were proposed as probable hosts of the virus during that time [20,21]. Moreover, a global sentiment analysis of social media posts identified fear as the dominant type of emotion when the disease was first detected, and the findings further show that such pandemic-specific anxiety was mainly attributable to a lack of COVID-19 screening tests and supplies of personal protection equipment [22].

This body of infodemiological research has advanced the literature by employing objective, innovative methods to elucidate public interest in several health topics during the pandemic. However, previous studies have not specified whether such information-seeking coping promotes or inhibits health behavior in the population. The present study extended this body of research by investigating the temporal associations between information-seeking coping and the vaccination rate during the initial COVID-19 vaccine rollout period.

### 1.2. Facilitating and Impeding Influences of Information-Seeking Coping on the Vaccination Rate

Since COVID-19 vaccines have become available to the public, vaccination anxiety has evolved as a new source of pandemic-specific fear among the general public [23]. Although large-scale, randomized controlled trials have demonstrated the efficacy of COVID-19 vaccines in mitigating infection risk [24], public opinion surveys have revealed mixed attitudes toward COVID-19 vaccination [25,26]. A certain proportion of survey respondents trusted the new vaccines and were eager to receive a vaccination; some felt unsure and reported uncertainty; and others expressed considerable vaccine hesitancy and skepticism. More specifically, a certain proportion of people with religious convictions are unwilling to get vaccinated due to ethical concerns regarding the use of fetal cell lines in the development of some COVID-19 vaccines [27,28]. As postulated by the uncertainty theory of anxiety, individuals tend to seek information in an attempt to mitigate their vaccination anxiety. However, coping theories state that the effects of coping may not always be beneficial [29,30].

In light of these theories, the present study aimed to distinguish between the facilitating and impeding influences of information-seeking coping on vaccination. The Internet constitutes a reservoir of diverse and potentially influential information. Information is frequently sought to increase one’s understanding of a matter or issue, and such knowledge enhancement is especially valuable for novel concerns when little is known. In the first wave of the pandemic, the potential facilitating effects of information-seeking coping were demonstrated by a large-scale survey [31]. The survey findings indicate that the respondents who perceived having adequate information about the pandemic tended to report a reduction in COVID-19 infection anxiety and sleep disturbance over time compared with those who perceived that they did not have enough information.

During the COVID-19 vaccine rollout, information seeking was common because unfamiliar modern technology was involved in developing some of the vaccines [32]. To curb the spread of the highly transmissible virus, COVID-19 vaccines were developed and officially approved for public use in an unprecedented short period of time. Timely dissemination of accurate information about these novel vaccines, such as how they were developed and approved, would be beneficial for improving transparency and resolving uncertainty among the general public. In the present study, information seeking was deemed to have a facilitating effect if the tendency of the public to seek information was positively associated with the vaccination rate during the vaccine rollout period.

Despite the advantages of information seeking, the Internet is also a hotbed of misleading and false information [33]. In the past decade, social media platforms have mushroomed, resulting in an escalation of public information sharing and exchanges with no major restrictions [34,35]. There has been a surge in the use of the Internet and social media in the initial wave of the COVID-19 pandemic, especially when the stay-at-home orders were in place [36,37]. After certain COVID-19 vaccines have been made available to the public, some skeptics have utilized social media as a propaganda tool to disseminate anti-vaccination disinformation and organize offline political actions, and these social media activities are found to exert negative impacts on public vaccination intent and coverage over time [38]. The easy accessibility to an avalanche of contradictory information regarding the COVID-19 pandemic has led to widespread confusion and anxiety worldwide [39,40]. The WHO has expressed immense concern about this information crisis and declared it to be an “infodemic” [41]. Processing a large amount of contradictory information may not satisfy an information consumer’s heightened epistemic needs but might actually generate greater uncertainty and confusion [42]. In the present study, information seeking was deemed to have an impeding effect if the tendency of the public to seek information was negatively associated with the vaccination rate during the vaccine rollout period.

### 1.3. Emergence of New COVID-19 Variants and the Vaccination Rate

Apart from the facilitating and impeding influences of information-seeking coping amid the COVID-19 vaccine rollout, we also propose that the magnitude of such influences may vary across time, probably due to various new viral variants arising [43]. As yet, the WHO has designated four SARS-CoV-2 variants—Alpha, Beta, Gamma, and Delta—as variants of concern [44]. The Beta variant was first detected in South Africa in May 2020, while the Alpha variant was first detected in the UK in September 2020; both were officially designated as variants of concern in December 2020. The Gamma variant was initially discovered in Brazil in November 2020 and was officially designated as a variant of concern in January 2021. Although the Delta variant was first identified in India in October 2020, it was officially designated as a variant of concern only in May 2021 when it began to spread rapidly throughout the world. Recent large-scale surveys have indicated that respondents were aware of the emergence of new variants during the various waves of the pandemic, and many expressed worries and concerns about the rising number of infections caused by these highly contagious COVID-19 variants [45,46].

Vaccination is the first-line measure in the COVID-19 mitigation plan of many countries, and health authorities worldwide have advocated that their population be vaccinated to prevent COVID-19 infection. While the global vaccination rate was low in the beginning of the rollout period, the pace of vaccination gradually accelerated in May and June 2021 [47]. Medical reports have indicated that the Delta variant has the highest infection rate, transmissibility, and symptom severity (e.g., the highest hospitalization risk) among the variants of concern [48]. Thus, we predict that the influences of information seeking will be especially prominent during the period in which the Delta variant is predominant.

### 1.4. Overview of the Present Study

The present study aimed to test the facilitating and impeding influences of information-seeking coping on the vaccination rate during the COVID-19 vaccine rollout period. These effects were tested using the objective, innovative method of Google Trends^TM^ analysis. This infodemiological method was used because Google has been the most used search engine for many years, accounting for more than 90% of the global search-related market share as of August 2021 [49]. Google Trends analysis is an ideal tool for investigating information-seeking behavior that reflects the extent to which people from a geographic area are concerned about certain issues.

In this study, we utilized Google Trends analysis to identify population-level interest in various COVID-19 variants by tracking the trajectory of Google search volumes on this topic. The associations between the search volume of information related to COVID-19 variants and the national vaccination rates were also investigated. The facilitating influence of information-seeking coping was operationalized as a positive association between Google search volumes and the national vaccination rate, whereas the impeding influence of information-seeking coping was operationalized as a negative association between search volumes and vaccination.

## 2. Materials and Methods

### 2.1. Study Design and Tool

The present study adopted a retrospective design using Google Trends analysis. Google Trends^TM^ is a freely accessible, Web-based tool for studying trends in the online search traffic of Google’s search engine, which reflects population-level information-seeking process and tendency [50]. This novel tool yields data that reflect Google users’ search interests, as indicated by the relative search volume (RSV). The RSV is relative in nature because the search volume has been adjusted to the number of Google users within a particular geographic region [51], thus facilitating comparisons in search popularity among different query terms. Approval from the board of research ethics was not required because this study involved open public data only.

### 2.2. Query Terms

Prior to the main study, an elicitation study was first conducted to obtain relevant query terms to include in the Google Trends analysis. In the elicitation study, participants were instructed to write as many keywords or phrases as possible when considering searching for information pertaining to COVID-19 variants using the Google search engine. Query terms given by more than 50% of the participants were finally included. The query terms pertaining to the Delta variant had the highest frequency (100%), followed by terms pertaining to COVID-19 variants in general (93%), the Beta variant (77%), and the Alpha variant (67%). Surprisingly, query terms pertaining to the Gamma variant were given by only 13% of the participants; therefore, this set of query terms was excluded from the present analysis.

As different Google users tended to search for the same topic using a variety of query terms, we first compared the RSVs among a set of similar query terms related to a particular COVID-19 variant. For each variant, only the most popular term was included in the data analysis to avoid redundancy. For instance, comparisons were made among the RSVs of query terms pertaining to the Delta variant (i.e., “*coronavirus delta*,” “*corona delta*,” “*covid delta*,” “*COVID-19 delta*,” and “*COVID-19 delta*”). The term “*covid delta*” had the highest RSV and was thus selected as the target query term. The same procedure was carried out for sets of similar query terms for the other COVID-19 variants included in this study. Based on these between-term comparisons, we finally included four target query terms in the Google Trends analysis: “*covid variant*,” “*covid alpha*,” “*covid beta*,” and “*covid delta.*”

### 2.3. Data Collection

**Figure 1:**
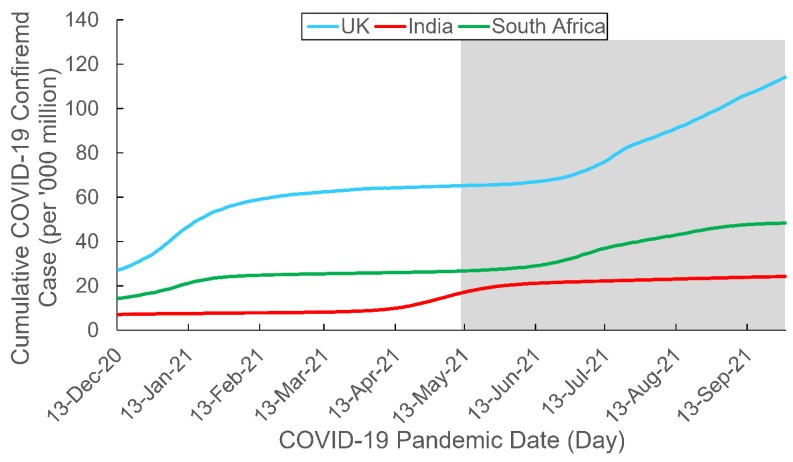
Cumulative COVID-19 confirmed case by country. The study period spanned from Epi Week 51 2020 (13–19 December 2020) to Epi Week 38 2021 (19–25 September 2021). The gray area depicts the Delta-predominant period. Data adapted from OurWorldInData.org, accessed on 6 June 2022.

**Figure 2:**
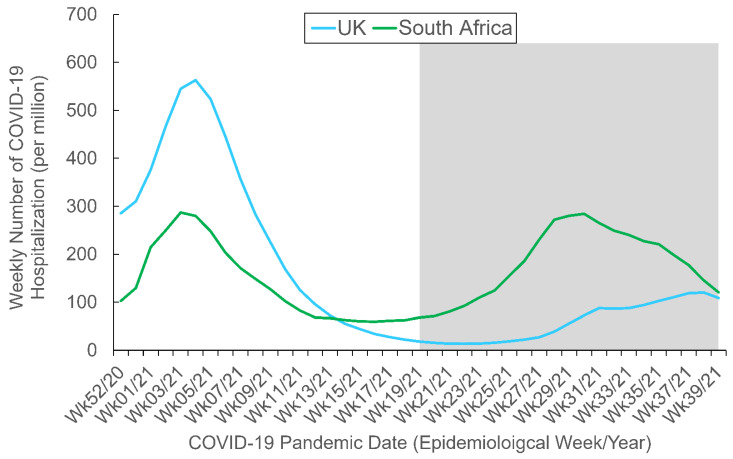
Weekly COVID-19 hospitalization by country. The study period spanned from Epi Week 51 2020 (13–19 December 2020) to Epi Week 38 2021 (19–25 September 2021). The gray area depicts the Delta-predominant period. Data adapted from OurWorldInData.org, accessed on 6 June 2022. The data for India are not available.

As the present study focused on the aforementioned three COVID-19 variants of concern, we retrieved data from the three countries in which one of the variants was first detected and were hardest hit by it—the UK, India, and South Africa [52]. Table 1 shows some demographic and socioeconomic data of these three countries of interest, and Figure 1 and Figure 2 depict the trajectories of the COVID-19 confirmed cases and hospitalization for the countries, respectively. The data collection period began on Epidemiological (Epi) Week 51 in 2020 (13–19 December 2020), when COVID-19 vaccines first became available for public use, and ended on Epi Week 38 in 2021 (19–25 September 2021).

### 2.4. Indicators of Interest

Population-level information-seeking coping was operationalized by the RSV. The RSVs of the four target query terms (“*covid variant*,” “*covid alpha*,” “*covid beta*,” and “*covid delta*”) were obtained from the Google Trends^TM^ website (https://trends.google.com, accessed on 27 September 2021). The RSV metric ranges from 0 to 100, with a higher value indicating a greater percentage of Google users in a particular geographic region who have searched for the target term over a designated date range. Hence, the target country and study period were entered when retrieving data for each country of interest.

National vaccination rate data were downloaded from the Our World In Data database [47]. In this study, the national vaccination rate was operationalized as the percentage of people who had received at least one dose of vaccine rather than the percentage of fully vaccinated people. Data on partial vaccination (i.e., having received at least one dose of COVID-19 vaccine) were used because South Africa had incomplete data on full vaccination, making between-country comparisons impossible. The Our World In Data database provides national vaccination data on a daily basis, while the Google Trends website provides search query data on a weekly basis. Hence, we computed the weekly averages for the national vaccination data to compare them with the weekly RSVs.

### 2.5. Data Analysis

To make between-country comparisons of the search queries for the COVID-19 variants, the RSVs of each query term in the three countries of interest were plotted on the same graph. Similar between-country comparisons were made for the national vaccination data.

As the RSV and vaccination rate were both time-series variables, pairwise cross-correlation analysis was performed to test the associations between search queries and the vaccination rate across multiple lagged values. The cross-correlation analysis identified the time lag(s) at which active search queries took place, and active search queries were operationalized as significant cross-correlations. Significant cross-correlation coefficients at negative lags indicated that high RSVs of the query term of a COVID-19 variant predicted a higher (positive value) or lower (negative value) vaccination rate. Significant cross-correlation coefficients at lag 0 indicated that RSVs coincided with vaccination rates. Significant cross-correlation coefficients at positive lags indicated that higher vaccination rates were associated with higher (positive value) or lower (negative value) subsequent RSVs.

All of the statistical analyses were performed with SPSS software version 26.0.0.0 (IBM Corporation, Armonk, NY, USA). Statistical significance was indicated by a *p* value less than 0.05.

## 3. Results

### 3.1. Between-Country Comparisons of the Vaccination Rate

**Figure 3:**
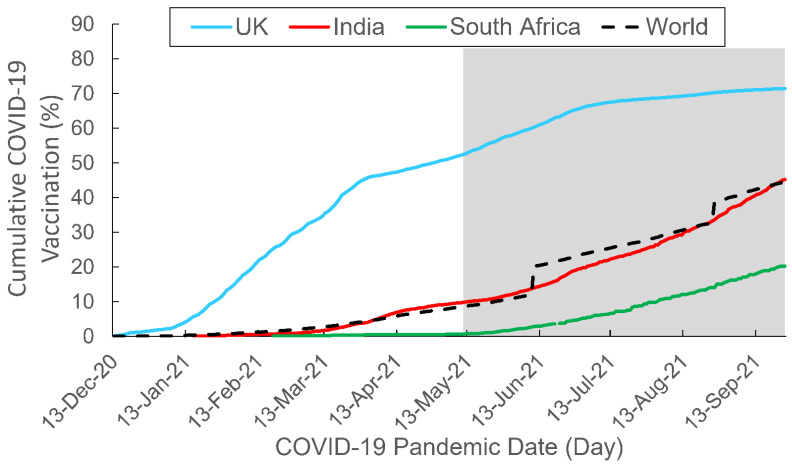
Cumulative COVID-19 vaccination rate by country. The study period spanned from Epi Week 51 2020 (13–19 December 2020) to Epi Week 38 2021 (19–25 September 2021). The gray area depicts the Delta-predominant period. Data adapted from OurWorldInData.org, accessed on 6 June 2022.

Figure 3 shows the cumulative COVID-19 vaccination rates among the three countries of interest, with the global data included as a benchmark. As shown in this figure, the growth in the UK vaccination rate approximated a convex upward curve, with the steepest rise in the percentage of vaccinated people occurring from the initial vaccine rollout until early April 2021 after which the curve begins to plateau. Between-country comparisons showed that the vaccination coverage in the UK was the highest among the three countries of interest throughout the study period. Furthermore, the increase in the vaccination rate in the UK was consistently greater than the global benchmark.

Unlike the UK curve, the Indian curve was much flatter and approximated a concave upward shape. The initial increase in the vaccination rate was comparatively mild, but the percentage showed a steeper climb during the Delta-predominant period. The Indian vaccination curve was similar to the global benchmark.

The South African curve remained quite flat throughout the vaccine rollout period. The initial increase in the vaccination rate was minimal, but the percentage slowly and steadily rose during the Delta-predominant period. The vaccination coverage in South Africa was the lowest among the three countries over the course of the study, and the increase in the vaccination rate was consistently lower than the global benchmark; the vaccination rate in South Africa was less than half of the global average throughout the study period.

### 3.2. Between-Country Comparisons of Google Search Trends

**Figure 4:**
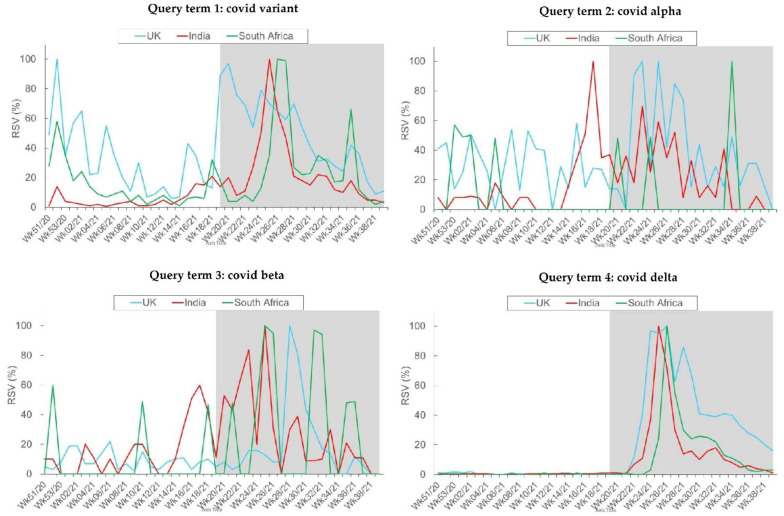
Weekly variations in relative search volumes (RSVs) of query terms related to COVID-19 variants by country. The study period spanned from Epi Week 51 2020 (13–19 December 2020) to Epi Week 38 2021 (19–25 September 2021). The gray area depicts the Delta-predominant period. Data adapted from the Google Trends^TM^ website.

Figure 4 depicts the RSV trend patterns for query terms related to COVID-19 variants during the study period in the three countries of interest. Overall, higher RSVs were found in the Delta-predominant period relative to the preceding period.

For the term “*covid variant*,” there were two marked RSV spikes (i.e., the highest volume of searches) in the UK: one spike occurred in late December 2020 and another in mid-May 2021. The RSV for this term peaked in mid-June and early July in both India and South Africa.

For the term “*covid alpha*,” the RSVs peaked at different periods in the three countries. The earliest RSV peak occurred at the end of April 2021 in India. In the UK, two spikes in the RSV for this term occurred in June, while in South Africa, the RSV peaked in late August.

For the term “*covid beta*,” the RSVs peaked around the same time in the three countries. All of these spikes were found within the Delta-predominant period and occurred from mid-June to early August 2021.

For the term “*covid delta*,” no queries were made before this variant was designated by the WHO as a COVID-19 variant of concern. During the Delta-predominant period, the RSVs were found to peak in mid-June to early July in all three countries of interest.

It is noteworthy that all such patterns of between-country differences in RSV trend were different from those of between-country differences in COVID-19 confirmed cases, COVID-19 hospitalization, and Internet penetration rate. These pattern differences suggested that public interest in COVID-19 variants of the populations was largely unaffected by these potential factors.

### 3.3. Time-Series Cross-Correlation between Google Search Queries and Vaccination Rates

The time-series cross-correlation analysis showed that the three countries differed in their patterns of temporal associations between the RSVs of COVID-19 variants and the vaccination rate. In the UK, all of the significant cross-coefficients were found at negative lags. Specifically, significant positive cross-correlations were found for the query terms “*covid beta*” and “*covid delta*” at Lag −16 (*r* = 0.66) and Lag −12 (*r* = 0.42), respectively. Conversely, significant negative cross-correlations were found for “*covid variant*” and “*covid delta*” at two later time points: Lag −5 (*r* = −0.35) and Lag −9 (*r* = −0.37).

Regarding India, all of the significant cross-coefficients were found at lags close to 0. Specifically, significant positive cross-correlations were found at Lag +1 for the following three query terms: “*covid variant*” (*r* = 0.60), “*covid beta*” (*r* = 0.36), and “*covid delta*” (*r* = 0.56). However, the only significant negative cross-correlation occurred at Lag +2 for “*covid beta*” (*r* = −0.44).

Regarding South Africa, the patterns of the three aforementioned terms were similar to those of the Indian data; however, the significant cross-correlations of the South African data showed a more dispersed distribution. Specifically, significant positive cross-correlations were found at Lag 0 for “*covid variant*” (*r* = 0.50) and “*covid beta*” (*r* = 0.44), but at Lags +5 and +9 for “*covid delta*” (*r*s = 0.66 and 0.46, respectively). There was also a significant negative cross-correlation at Lag +2 for “*covid beta*” (*r* = −0.43), a finding similar to that from the Indian data. In addition, two significant positive associations were found at Lag −3 and Lag +11 for “*covid alpha*” (*r*s = 0.45 and 0.44, respectively), but no such findings were found for the other two countries.

## 4. Discussion

By adopting Google Trends analysis, the present study examined the temporal associations between public interest in various COVID-19 variants and the national vaccination rates during the vaccine rollout period. There was considerable public interest in various COVID-19 variants in the three countries initially affected by these variants, namely, the UK, India, and South Africa. The trajectories of the search trends reflect longitudinal changes in such interest. Notably, there was a larger volume of search queries during the Delta-predominant period than before Delta became predominant. Such temporal distinctions may reflect variations in the nature of the different COVID-19 variants. Compared with the other variants, the Delta variant has caused a greater number of COVID-19 infections and hospitalizations, and this highly contagious variant has spread more rapidly and extensively throughout the world [53]. Hence, public interest in understanding the various COVID-19 variants increased during the Delta-predominant period.

More importantly, this study is the first to investigate the associations between search queries and behavioral data regarding the preventive measure of COVID-19 vaccination. The findings demonstrate that information-seeking coping is a double-edged sword, which can be facilitating or impeding regarding vaccination. Search queries of COVID-19 variants are positively related to the vaccination rate, probably because information consumers’ epistemic needs have been satisfied and they tend to be relieved of their uncertainty [54]. However, search queries of COVID-19 variants are also found to be negatively related to the vaccination rate, probably due to problems arising from information overload and perceptual biases [31]. Furthermore, these two hypothetical effects are not equivocal. A close examination of the present data reveals that the facilitating and impeding effects of information-seeking coping tend to differ in their predominance and occurrence throughout the vaccine rollout period, as discussed below.

### 4.1. Predominance of Positive Search Query–Vaccination Associations

This promising infodemiological method generates novel findings that contribute to the literature by revealing two major empirical patterns. The first pattern is that the majority of the significant associations are positive. These positive associations indicate that a greater public interest in topics related to COVID-19 variants may be associated with higher national vaccination rates. Notably, the positive time-series associations are found at different time points in the three countries of interest. In the UK, all of the significant positive associations between the search queries and the vaccination rate occurred before the Delta variant became predominant and when the national vaccination rate began to sharply rise. The UK vaccination rate was the highest among the three investigated countries and was consistently higher than the global average throughout the study period.

In India, the significant positive associations between public interest in the various COVID-19 variants and the vaccination rate coincided at around the same time, but at a much later period than in the UK. The vaccination curve of India shows a similar trend; the curve is rather flat at the beginning of the vaccine rollout and gradually rises halfway through the study period. The vaccination rate in India was similar to the global benchmark amid the study period.

In South Africa, some of the significant positive associations were similar to those found in India because the search queries of the COVID-19 variants and the vaccination rates in the two countries coincided. However, some of the significant positive associations occurred at much later periods in South Africa than in the other two countries. Similar to India, South Africa’s vaccination rate was very low at the outset of the vaccine rollout period and then slowly increased throughout the Delta-predominant period. South Africa had the lowest vaccination rate among the three investigated countries, and it was consistently below the global average throughout the study period. In summary, such between-country time-series variations were consistent with the temporal changes in the vaccination rate and pace in the three countries of interest.

### 4.2. Occurrence of Positive Search Query–Vaccination Associations Prior to Negative Associations

The second major pattern is that although significant positive associations are more common, significant negative associations are also found. Interestingly, these few negative associations occurred at later time points than the significant positive associations. Such temporal distinctions may reflect the diverse types of information disseminated throughout the vaccine rollout period, and such information might have affected people’s intention to vaccinate. In a nationally representative survey conducted in the UK before the vaccine rollout [55], a considerable proportion of respondents expressed their intention to vaccinate, but concerns about vaccine safety and side effects were major obstacles that weakened this intention for some respondents. A few months after the initiation of vaccine rollout, the same concerns regarding vaccination risks were expressed in social media posts among Indian residents [56]. A public opinion poll posted on Twitter shortly after the rollout has also revealed mixed attitudes towards vaccine safety: half of the respondents expressed trust, while the other half felt unsure [57]. Moreover, thematic analyses on social media posts have similarly indicated that COVID-19 vaccine safety and efficacy concerns were the most highly discussed topic during the first wave of the pandemic [58,59].

The efficacy and safety of some of the newly developed vaccines have been demonstrated in large-scale, randomized controlled trials [24,60]. For instance, Novavax Inc. has announced that its vaccine is highly effective in protecting against the original COVID-19 strain as well as the other variants in both UK and South African samples [61]. In addition, a recent review of the various COVID-19 vaccines indicates that the side effects are mild to moderate in severity and are not fatal [62]. As the vaccine rollout proceeds, however, sporadic cases of vaccination-related deaths and fatal side effects have been reported in the mass media as an increasing number of people have been vaccinated [63,64]. These rare but vivid, fearful incidents tend to create long-lasting mental impressions and thus more readily come to mind in decision making. This type of perceptual bias has been well documented in the cognitive science literature and is called the availability heuristic [65].

As information consumers tend to rely on heuristic information processing when confronting novel risks, they are especially vulnerable to the availability heuristic, which often leads to misjudgment [42,66]. Applying this common perceptual bias to the context of COVID-19 vaccination, public vaccine hesitancy may grow if information regarding the rare cases of deaths or severe adverse physical reactions is widely available [67]. As this fear-inducing information accumulates, people may process a greater volume of such information, which may elicit greater vaccination anxiety and hesitancy. Hence, the impeding influence of information-seeking coping is generally found at later time points in the vaccine rollout period.

### 4.3. Recommended Information Strategies for Combating the COVID-19 Infodemic

Given that information-seeking coping during the COVID-19 vaccine rollout tends to exert both desirable and undesirable influences, there should be a greater effort to maximize its facilitating impact while minimizing its impeding impact on vaccination uptake when designing health education programs to combat the infodemic. As physical distancing measures were imposed for an extended period in many countries, the Internet has played a major role in public health education during the pandemic. Sentiment analysis indicates that although the general public has expressed considerable anxiety and panic during the initial phase of the COVID-19 pandemic, public sentiment toward vaccination tends to become more positive and public trust tends to grow as greater knowledge and scientific findings are available over time [58]. Recent findings have shown that the type of information provider plays a key role in improving the accuracy and attitude of public beliefs during a health crisis, with government agencies and news media perceived as being more reliable than social peers [68].

To combat the infodemic, public health authorities and news media can work together to maximize the facilitative impact of information-seeking coping by combining biomedical expertise with vivid media presentations that capture the public’s attention. Some scholars have encouraged healthcare professionals to become acquainted with social media platforms to leverage greater public trust [35,59,69]. Specifically, some popular platforms (e.g., Twitter, Facebook) can be utilized by these professionals for timely communication of scientifically sound medical evidence regarding vaccine content and safety. Multiple health promotion strategies can be adopted, including public health messaging, infographics, and online awareness campaigns for raising public attention and engagement. In addition, healthcare professionals can hold interactive sessions inviting community members who have recovered from COVID-19 infection to share their firsthand experiences in the treatment and recovery processes [35]. In addition, information technology experts should combat fake news and disinformation related to anti-vaccination issues through a multi-prong strategy, such as artificial intelligence programs for detecting and filtering misinformation and public education on responsible use of the Internet and social media [38,69].

A more systematic approach, namely the WHO’s 3Cs model, could also be adopted as a blueprint when preparing health information for online dissemination [70]. This model states that vaccine intention can be boosted by addressing the issues of *confidence*, *complacency*, and *convenience*. Thus, the design of Web-based health education programs could focus on these three factors. First, public confidence in vaccine safety can be strengthened by citing relevant statistics to weaken the influence of the availability heuristic; for example, national statistics reveal real-life data showing that cases of vaccine-induced fatality are indeed rare [71]. Myth busting through gamification techniques is another efficacious strategy for weakening the availability bias [72].

Second, public complacency regarding the low perceived risk of infection can be mitigated by emphasizing the high transmissibility of the COVID-19 variants. Interactive simulator is a promising data visualization strategy that can vividly present health information to capture the public’s interest [73]. A good example of interactive simulation is the online animated simulation on the Washington Post website that uses the billiard ball model to illustrate the exponential growth of COVID-19 cases [74]. Increasing the information salience through adopting such innovative, eye-catching approaches can foster recall of the health information highlighted in health promotion campaigns, thereby reducing the availability bias.

Third, convenience can be enhanced by making health education websites more visible and searchable because search engines have emerged as indispensable tools for information consumers when facing novel issues [75]. In addition, several cloud-computing-based Web features can be utilized to make information searching more convenient [76]. For instance, the nearest vaccine locations can be easily searched and located, and vaccine appointments can be downloaded into Google calendar or MS Outlook for easy viewing and reminder setup [77]. Taken together, guided by the WHO’s 3Cs model, these three clusters of Web strategies can be utilized in campaigns designed to decrease public vaccine hesitancy and enhance adherence to public health recommendations.

### 4.4. Research Limitations and Future Research Directions

The present study is the first to examine the temporal associations between public interest in various COVID-19 variants and national vaccination rates; however, some limitations must be considered when interpreting the findings. First, the search query terms are limited to specific COVID-19 variants. Apart from COVID-19 variants of concern, several variants of interest have also been designated by the WHO, such as the Iota variant first identified in the US and the Lambda variant first identified in Peru [44]. These COVID-19 variants of interest are not included because none of the respondents mentioned them in the elicitation study. As the virus characteristics and societal impact of COVID-19 variants of interest and COVID-19 variants of concern differ considerably, the present findings derived from the variants of concern may not necessarily be generalizable to the variants of interest. Future research may expand the scope to include a greater variety of COVID-19 strains to achieve a more nuanced analysis of their effects.

Second, the present study investigated the time period during which several newly developed COVID-19 vaccines are first rolled out to the public. At the time of writing, the COVID-19 pandemic continues to evolve without any clear sign of ceasing. Other vaccination strategies (e.g., nasal spray) are currently being tested, and additional COVID-19 variants may be discovered with time [78,79]. Thus, research should be conducted to replicate this study design in future waves of new COVID-19 mutations and to further investigate the effects of information seeking across a longer time span.

Finally, although Google Trends analysis can overcome the major methodological problems faced by self-report survey methods, subjective data regarding users’ experiences are largely unknown. The underlying motivation of information seeking and the content of the information sought can provide further insights into individual differences in information-seeking coping and its effectiveness. Hence, there is still value in conducting self-report studies that yield subjective data, and future studies may adopt a mixed-methods approach for a more comprehensive investigation of information-seeking coping and its association with vaccination.

## 5. Conclusions

By adopting an infodemiological approach, the present Google Trends analysis identified temporal associations between public interest in COVID-19 variants and national vaccination rates during the vaccine rollout period. Information-seeking coping was found to be a double-edged sword that has both benefits and impediments during this period. The facilitative influences on vaccination are more prominent and tend to emerge at earlier time points, while the few impeding influences tend to occur later.

As the Internet and social media platforms have emerged as main sources of information when COVID-19-related physical distancing measures are prevalent, public health authorities, information technology sector, and news media may collaborate to strengthen the efficacy of Web-based health promotion campaigns to maximize the facilitative influences of information-seeking coping on vaccination uptake to combat the infodemic more effectively. Specifically, healthcare professionals may collaborate with social media and utilize multiple strategies (e.g., public health messaging, infographics, and online interactive sharing sessions) for enhancing public awareness and engagement behavior toward COVID-19 vaccination. In addition, algorithmic solutions may be used to identify and filter out false news and misinformation. Moreover, health promotion campaigns should also focus on enhancing public knowledge on not only vaccine content and safety but also responsible Internet use and media literacy (e.g., fact checking and debunking).

## Figures and Tables

**Table 1:** 2021 Demographic and socioeconomic data of the three countries of interest.

Country	Nominal GDP per Capita ^1^	Estimated Population ^2^	Number of Internet Users ^2^	Internet Penetration Rate ^2^	Google Market Share ^3^
UK	USD 46,200	66,959,016	63,544,106	95%	92%
India	USD 2,116	1393,409,038	755,820,000	54%	99%
South Africa	USD 6,861	60,041,994	34,545,165	58%	92%

^1^ Source: statisticstimes.com; ^2^ Source: internetworldstats.com; ^3^ Source: gs.statcounter.com.

## Data Availability

The data presented in this study are available on request from the author.
